# Operations on Hysterical Patients

**Published:** 1898-03-26

**Authors:** 


### OPERATIONS ON HYSTERICAL PATIENTS.

We would not for the world have people imagine that we scoff at modern surgery; still, the necessity for caution and for moderation in surgical suggestions is every week more strongly borne in upon us by what we find to be going on in different parts of the world. Here is a little history which we take from a lecture delivered by Dr. Joseph Bryant, professor of surgery, at the Bellevue Hospital Medical College, and reported in the *Medical News* of New York for March 3rd: A female, about 35 years of age, was referred to him for diagnosis and treatment. She had suffered from persistent and annoying pains in the pelvic, right inguinal, and lumbar regions for a long time. For the relief of this the left ovary had been removed, and nephrorrhaphy on the right side had been practised some time before. Inasmuch as there only remained the appendix, the uterus, and the right ovary, the professor's attention was naturally directed to these parts and to their condition. No evidence of ovarian, uterine, or broad ligament disease could be found, but a careful palpation disclosed the seat of the appendix; its extent apparently increased in size, and the presence on moderate pressure of the pain which had throughout characterised her affliction; in fact, she exclaimed, "That is the same kind of pain I have had all the time." Then the appendix was removed, and certain lesions were found in it; but the pain continued the same as before! Since then the patient has submitted to the removal of the right ovary also; but still she is tormented with the pain, and still she is anxious for further effort to relieve it! With stories like this before us we need not hesitate to urge caution in regard to modern extensions of the field of operative surgery.

